# Mass Spectrometry Analysis of Hepcidin Peptides in Experimental Mouse Models

**DOI:** 10.1371/journal.pone.0016762

**Published:** 2011-03-08

**Authors:** Harold Tjalsma, Coby M. M. Laarakkers, Rachel P. L. van Swelm, Milan Theurl, Igor Theurl, Erwin H. Kemna, Yuri E. M. van der Burgt, Hanka Venselaar, Bas E. Dutilh, Frans G. M. Russel, Günter Weiss, Rosalinde Masereeuw, Robert E. Fleming, Dorine W. Swinkels

**Affiliations:** 1 Laboratory of Genetic, Endocrine and Metabolic Diseases, Department of Laboratory Medicine, Radboud University Nijmegen Medical Centre, Nijmegen, the Netherlands; 2 Department of Pharmacology and Toxicology, Radboud University Nijmegen Medical Centre, Nijmegen, the Netherlands; 3 Department of Internal Medicine, Clinical Immunology and Infectious Diseases, Medical University of Innsbruck, Innsbruck, Austria; 4 Department of Ophthalmology and Optometry, Innsbruck Medical University, Innsbruck, Austria; 5 Biomolecular Mass Spectrometry Unit, Department of Parasitology, Leiden University Medical Centre, Leiden, the Netherlands; 6 Centre for Molecular and Biomolecular Informatics (NCMLS), Nijmegen, the Netherlands; 7 Department of Pediatrics, St. Louis University Liver Center, St. Louis University School of Medicine, St. Louis, Missouri, United States of America; Charité-University Medicine Berlin, Germany

## Abstract

The mouse is a valuable model for unravelling the role of hepcidin in iron homeostasis, however, such studies still report *hepcidin* mRNA levels as a surrogate marker for bioactive hepcidin in its pivotal function to block ferroportin-mediated iron transport. Here, we aimed to assess bioactive mouse Hepcidin-1 (Hep-1) and its paralogue Hepcidin-2 (Hep-2) at the peptide level. To this purpose, fourier transform ion cyclotron resonance (FTICR) and tandem-MS was used for hepcidin identification, after which a time-of-flight (TOF) MS-based methodology was exploited to routinely determine Hep-1 and -2 levels in mouse serum and urine. This method was biologically validated by hepcidin assessment in: i) 3 mouse strains (C57Bl/6; DBA/2 and BABL/c) upon stimulation with intravenous iron and LPS, ii) homozygous *Hfe* knock out, homozygous transferrin receptor 2 (Y245X) mutated mice and double affected mice, and iii) mice treated with a sublethal hepatotoxic dose of paracetamol. The results showed that detection of Hep-1 was restricted to serum, whereas Hep-2 and its presumed isoforms were predominantly present in urine. Elevations in serum Hep-1 and urine Hep-2 upon intravenous iron or LPS were only moderate and varied considerably between mouse strains. Serum Hep-1 was decreased in all three hemochromatosis models, being lowest in the double affected mice. Serum Hep-1 levels correlated with liver *hepcidin-1* gene expression, while acute liver damage by paracetamol depleted Hep-1 from serum. Furthermore, serum Hep-1 appeared to be an excellent indicator of splenic iron accumulation. In conclusion, Hep-1 and Hep-2 peptide responses in experimental mouse agree with the known biology of hepcidin mRNA regulators, and their measurement can now be implemented in experimental mouse models to provide novel insights in post-transcriptional regulation, hepcidin function, and kinetics.

## Introduction

The control of iron homeostasis acts at both the cellular and the systemic level and involves a complex system of different cell types, transporters and signals. To maintain systemic iron homeostasis, communication between cells that absorb iron from the diet (duodenal enterocytes), consume iron (mainly erythroid precursors) and store iron (hepatocyte and tissue macrophages) must be tightly regulated. The recently identified β-defensin-like anti-microbial peptide hepcidin is thought to be the long anticipated regulator that controls iron absorption and macrophage iron release. It is synthesized in the liver upon changes in body iron stores, anemia, hypoxia and inflammation, and secreted in the circulation [1]. Hepcidin is reported to counteract the function of ferroportin, a major cellular iron exporter protein in the membrane of macrophages and the basolateral site of enterocytes, by inducing its internalization and degradation [2].

Much of the data concerning the involvement of hepcidin in iron metabolism were initially generated in mouse models. Whereas humans, rats, pigs and dogs have a single gene, due to gene duplications there are 2 hepcidin genes in mice, i.e. *hepcidin-1* and *hepcidin-2* [2], [3]. These two genes are located in the same region on mouse chromosome 7. While *hepcidin-1* is almost exclusively expressed in the liver, *hepcidin-2* is also expressed in the pancreas [4], [5].

At the functional level both *hepcidin* genes are upregulated in iron loaded mice [5]. Induction of inflammation increased *hepcidin-1* [6], but repressed *hepcidin-2* in the liver [4], [7]. Targeted disruption of the *hepcidin-1* gene has been shown to result in severe tissue iron overload [8], whereas mice overexpressing *hepcidin-1* develop iron deficiency anemia [6]. However, mice overexpressing *hepcidin-2* presented with normal iron metabolism [4]. This suggests that only mouse hepcidin-1 (Hep-1) peptide is able to regulate iron homeostasis and highlights the non-redundant roles of Hep-1 and Hep-2 in mice and points toward divergent functions of both peptides. The evolutionary advantage of having two hepcidin proteins is, however, still unknown.

Both peptides are 68% identical and contain 8 cysteine residues, which form 4 intra-molecular disulfide bridges in the 25-amino-acid (aa) mature peptide (predicted structures are shown in Figure S1). While Hep-1 is most similar to human Hepcidin-25, which has a proven role in iron metabolism, Hep-2 shares some common features with fish hepcidin-like peptides and therefore might share common functions with the latter peptides, possibly in innate immunity [9], [10].

As the mouse has been shown to be a valuable model to unravel iron metabolism disorders, the current study aimed at the development of a methodology to measure mouse hepcidin on the peptide level by mass spectrometry. For reasons of comprehensiveness, we aimed at the analysis of both Hep-1 and Hep-2 in mouse serum and urine samples, despite the fact that the exact function of the latter peptide (if any), needs to be elucidated.

## Results

### Identification of serum Hep-1 and urine Hep-2

**Figure 1:**
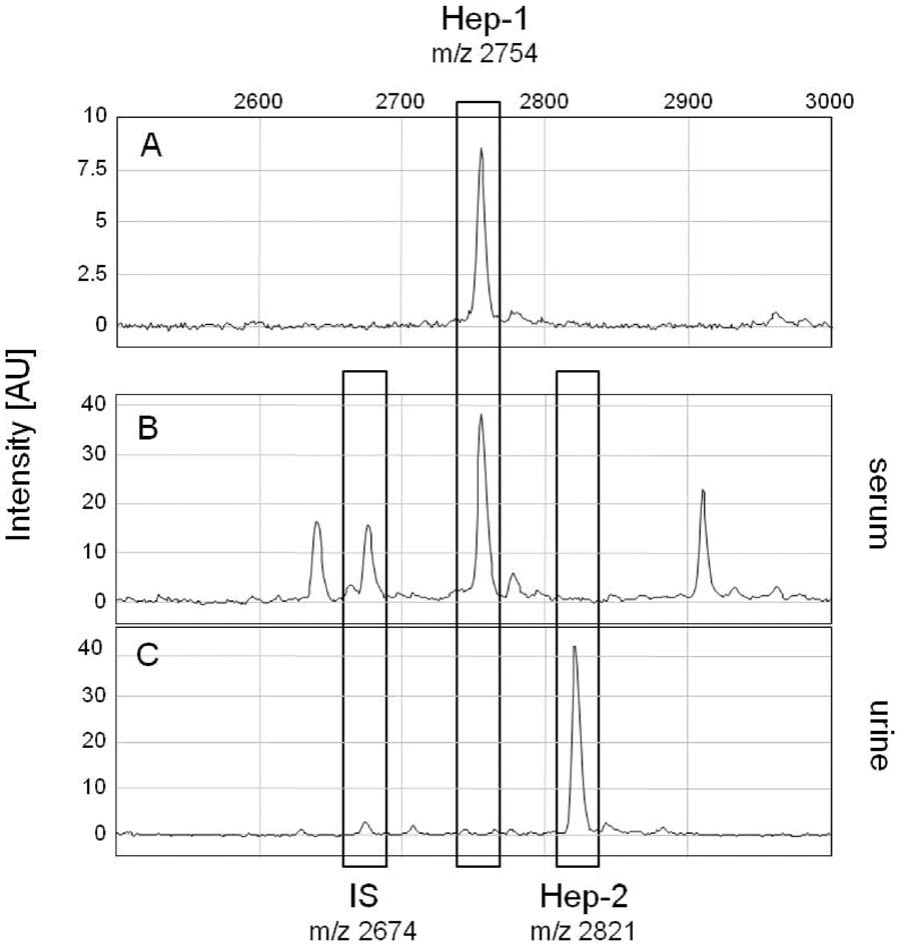
IMAC-Cu^2+^ TOF MS analysis of mouse Hepcidin. ***A***. Synthetic mouse Hep-1 peptide; ***B.*** Serum from a C57Bl/6 mouse and ***C.*** urine from a FVB mouse (***C***) spiked with Hepcidin-24 [13] as internal standard (IS). Positions of Hep-1, Hep-2 and IS are indicated. Peak intensity is given in arbitrary units (AU).

IMAC-Cu^2+^ on-chip chemistry was used to enrich hepcidin peptides from mouse serum and urine after which peptides were visualized by TOF MS. A serum-derived peak with measured mass of m/z 2754 matched to the synthetic Hep-1 reference peptide, whereas an urine-born peak with m/z 2821 corresponded to the theoretical mass of Hep-2 (Figure 1). Upon chemical reduction, the presumed Hep-1 and Hep-2 peaks displayed a mass shift of 8 Da, which is diagnostic of 4 disulfide bridges in the parental peptides (Figures S2 and S3) [11]. Conclusively, tandem MS analysis on a mouse urine sample as performed previously for human urine hepcidin [12], [13] showed that the suspected Hep-2 peak indeed matched with the deduced amino acid sequence of the *hepcidin-2* gene (Figure S4). For the identification of Hep-1 an accurate mass measurement using MALDI-FTICR MS in combination with a bioinformatics approach was employed. Searches in the mouse protein database resulted in 6 possible peptides containing 8 cysteine residues within a mass tolerance window of ±50 ppm from Hep-1 (Table S1). External calibration of the MALDI-FTICR spectrum (Figure S5) resulted in a monoisotopic mass measurement of *m/z* 2753.032 with a standard deviation of ±2.5 ppm. From this it was deduced that only the masses of Hep-1 and a 28 aa internal peptide from metallothionein-1 fitted within three standard deviations (>99.7% confidence) of the measured mass of this peak. Interestingly, further investigation of the MALDI-FTICR spectrum showed a monoisotopic signal at m/z 2637.989 that could be derived from an in-source peptide fragmentation event. This was evident from the isotopic distributions of the signals at 2753 and 2637 that were close to identical, implying similar aa compositions. Based on the mass difference, the m/z 2637.989 signal could only be assigned to the Hep-1 peptide lacking the N-terminal aspartic acid (D) residue (Figure S5). From these combined analyses it was concluded that the employed on-chip chemistry is an convenient approach to detect mouse hepcidin peptides.

### Hep-1 and -2 peptide levels in serum and urine from different mouse strains

The broad applicability of the IMAC-Cu^2+^ TOF MS procedure was illustrated by the detection of Hep-1/-2 peptides in serum and urine from FVB, DBA/2, C57Bl/6 and BALB/c mice (see Figures 1 and S6), Hep-1 appeared to be the dominant hepcidin form in serum, whereas Hep-2 was dominant in urine. Notably, Hep-1 and -2 from DBA/2 mice have a different mass due to asparagine to lysine (Hep-1) and serine to phenylalanine (Hep-2) substitutions at positions 16 and 18 as compared to the respective mature hepcidin peptides (see Figure S1). The observed deviate masses of Hep-1 (m/z 2768) and Hep-2 (m/z 2881) of DBA mice match to their altered aa composition. Similar to humans, a presumed amino-terminally truncated 20 aa isoform of Hep-2 was detected in urine of all mouse strains, whereas possible 22 aa and 23 aa isoforms of Hep-2 were detected in urine from C57Bl/6 and DBA/2 mice. Isoform levels appeared highest for DBA/2 mouse (Figure S7), however, it should be realized that no MS/MS confirmation of the identity of these Hep-2 isoforms was obtained. Together, these experiments show that Hep-1 and Hep-2 (and possible isoforms) can be accurately detected in serum and urine from different mouse strains.

### Serum Hep-1 is predominantly produced by the liver

**Figure 2:**
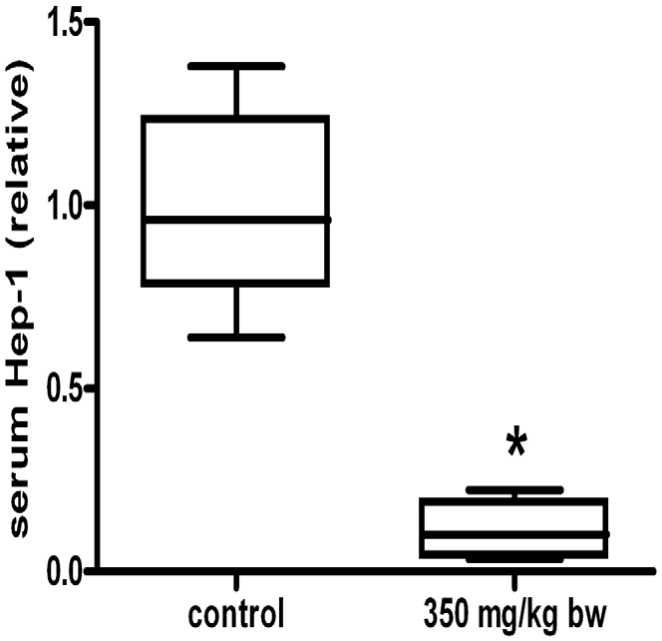
Serum Hep-1 levels in control FVB mice (n = 5) and mice 24 hrs after i.p. treatment with a sublethal hepatotoxic dose of 350 mg paracetamol/kg bw (n = 4). Data are depicted as lower quartile, median and upper quartile (boxes), and minimum and maximum ranges (whiskers). *Differences between control and treated mice tested by (two tailed) Mann-Whitney test (*P*<0.05).

To further gain *in vivo* evidence that Hep-1 is a liver-produced peptide, FVB mice were treated with a sub-lethal dose of paracetamol to induce acute liver damage. This clearly showed that after 24 hrs, serum Hep-1 significantly decreased (Figure 2), whereas serum ALAT levels significantly increased from mean 52 U/L (SD 17) in controls to a mean of 11188 U/L (SD 11823) in treated mouse. The latter is diagnostic for severe liver damage. In contrast, urine Hep-2 levels (collected until 24 hours after start of treatment) remained essentially unaffected during treatment which is suggestive for expression in other organs (data not shown).

### Correlation of serum Hep-1 with liver *hepcidin-1* mRNA and liver/spleen iron content

**Figure 3:**
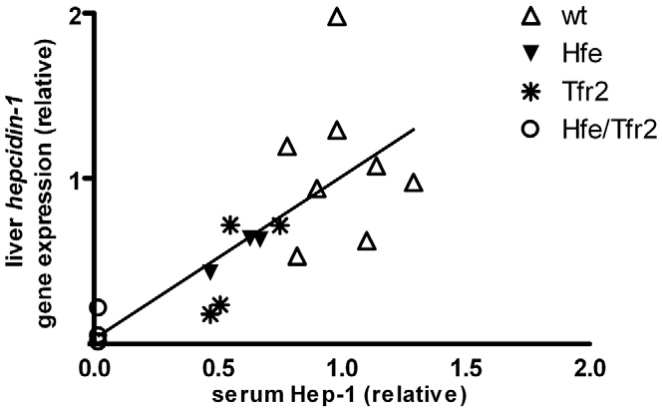
Correlation between serum Hep-1 and liver *hepcidin-1* mRNA. Data are obtained from wild type mice (n = 8), Hfe knockout mice (n = 3), TfR2 ^Y245X^ mice and double affected Hfe/TfR2 ^Y245X^ mice (n = 4). Real time values are expressed as mRNA content relative to the mean value obtained for the wild-type mice. Likewise serum Hep-1 values are expressed relative to the mean value from the wild type mice. r (Spearman) = 0.8263, *P<0.001.*

**Figure 4:**
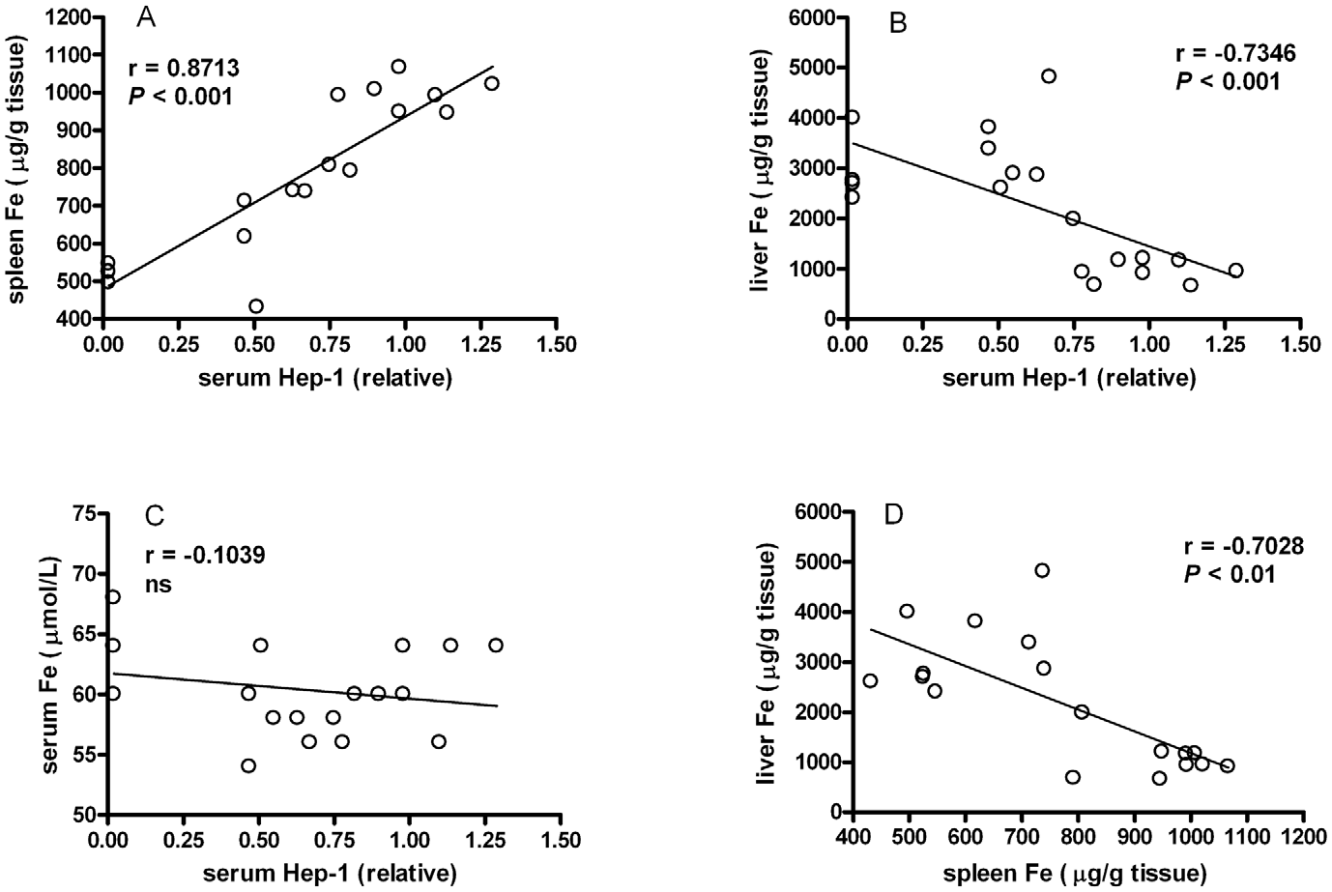
Correlations between serum Hep-1 and liver *hepcidin-1* mRNA, and iron concentrations of serum, spleen and liver. Data are obtained from wild type mice (n = 8), Hfe knockout mice (n = 3), TfR2 ^Y245X^ mice and double affected Hfe/TfR2 ^Y245X^ mice (n = 4). Correlation was tested by Spearman. ns, not significantly different.

As the current study provides a new tool to directly evaluate bioactive Hep-1 levels in the circulation, we aimed to assess the correlation between Hep-1 and liver *hepcidin-1* gene expression. We also included spleen iron levels in the correlations to assess whether hepcidin induced iron withholding by the reticulo-endothelial macrophages. Therefore, serum, spleen and liver tissue from control mouse and hemochromatosis mouse models were collected for parallel analysis of these parameters. As shown in Figure 3, serum Hep-1 levels correlated significantly with liver *hepcidin-1* mRNA levels (r = 0.8263,p<0.001). Interestingly, serum Hep-1 levels showed to be an excellent determinant for spleen iron levels (Figure 4A). Furthermore, liver iron levels, but not serum iron concentrations, significantly correlated with serum Hep-1 levels (Figure 4B and C).

### Hepcidin levels in hemochromatosis mouse models

**Figure 5:**
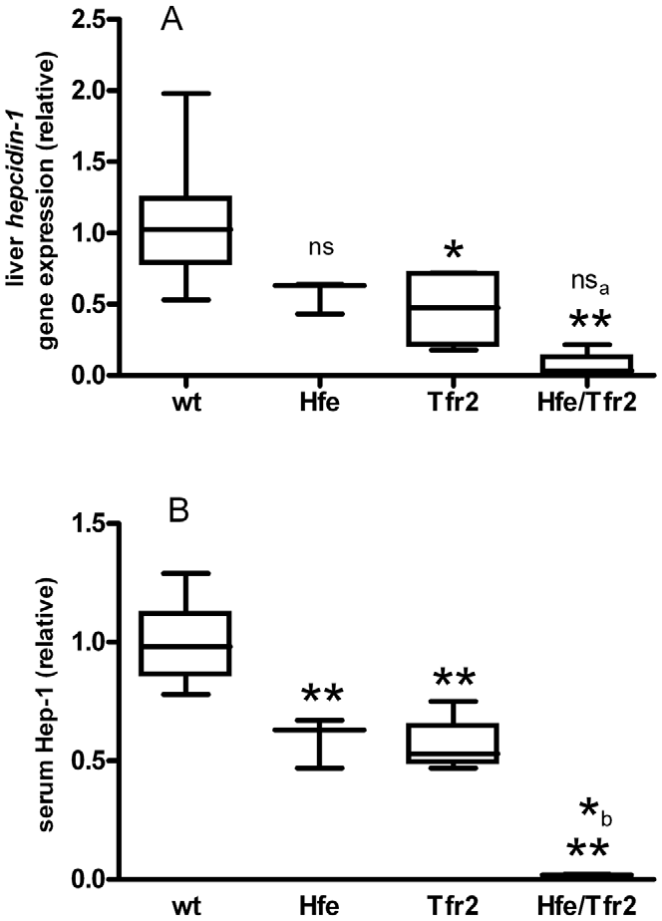
Serum Hep-1 levels and liver *hepcidin-1* expression obtained for mouse models of hereditary hemochromatosis relative to wild-type (wt) mouse. Data are depicted as lower quartile, median and upper quartile (boxes), and minimum and maximum ranges (whiskers). Statistical analysis were performed using ANOVA and Dunnett's comparisons. Ns, not significantly different; *, *P*<0.05; **, *P*<0.01; all compared to wild-type; a and b, significancy of difference to homozygous Tfr2 mutated mice.

To evaluate the functional importance of measured Hep-1 and Hep-2 levels in various genetic variants of hemochromatosis, serum was collected from wild-type mice (wt), Hfe KO, Tfr2^y245x^ or Hfe/TfR2^y245x^ mouse. These analyses showed that Hep-2 levels in serum were below the lower limit of detection. As shown in Figure 5, liver *hepcidin-1* mRNA levels (panel A) as well as serum Hep-1 levels (panel B) were decreased in all three hemochromatosis models and lowest in the double affected *Hfe/TfR2^ y245x^* mice.

### Hep-1 and Hep-2 levels upon challenge with iron and LPS

**Figure 6:**
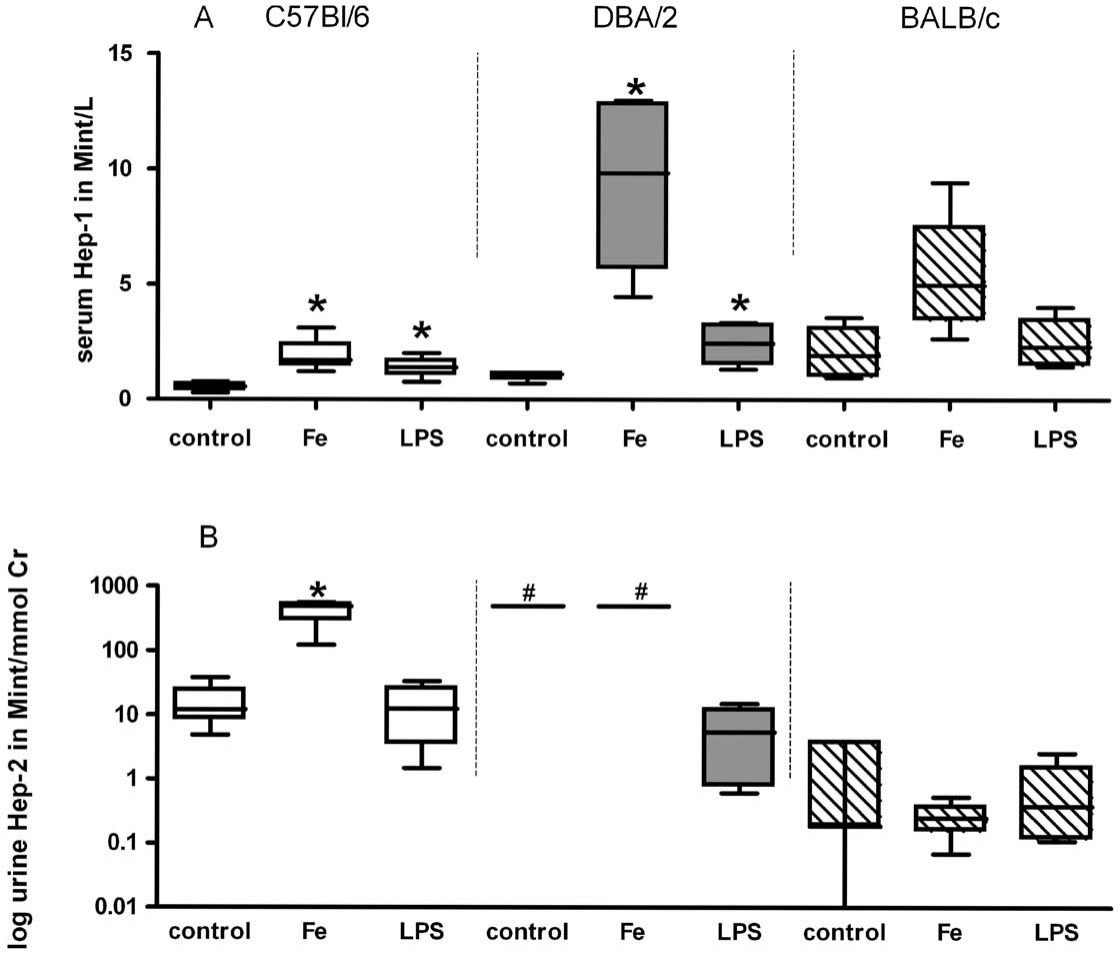
Serum and urine hepcidin levels upon stimulation by iron and LPS. Hep-1 in serum in Mega-intensities (Mint) per L (Mint/L) at baseline (control, n = 4) 24-hours after iron administration (n = 4) and LPS stimulus (n = 4). Hep-2 in urine in Mint/mmol creatinine (Cr) at baseline (n = 4) and 24-hours after iron (n = 4) and LPS (n = 4) stimulus. #, Hep-2 values in control and Fe group DBA/2 are >500 Mint/mmol Cr. Urine sample restriction did not allow more precise measurements. Therefore, *P*-values for urine Hep-2 were not calculated for DBA/2 mice. C57Bl/6, open bars; DBA/2, closed bars; BALB/c mice, dashed bars. Data are depicted as lower quartile, median and upper quartile (boxes), and minimum and maximum ranges (whiskers). Differences of iron and LPS treated mouse from control within mouse strain are tested by Whitney tests; *, *P*<0.05. In this specific experiment, hepcidin levels were measured in the absence of IS and therefore expressed in Mint/L for serum or/mmol Creatinine (Cr) for urine [12].

**Figure 7:**
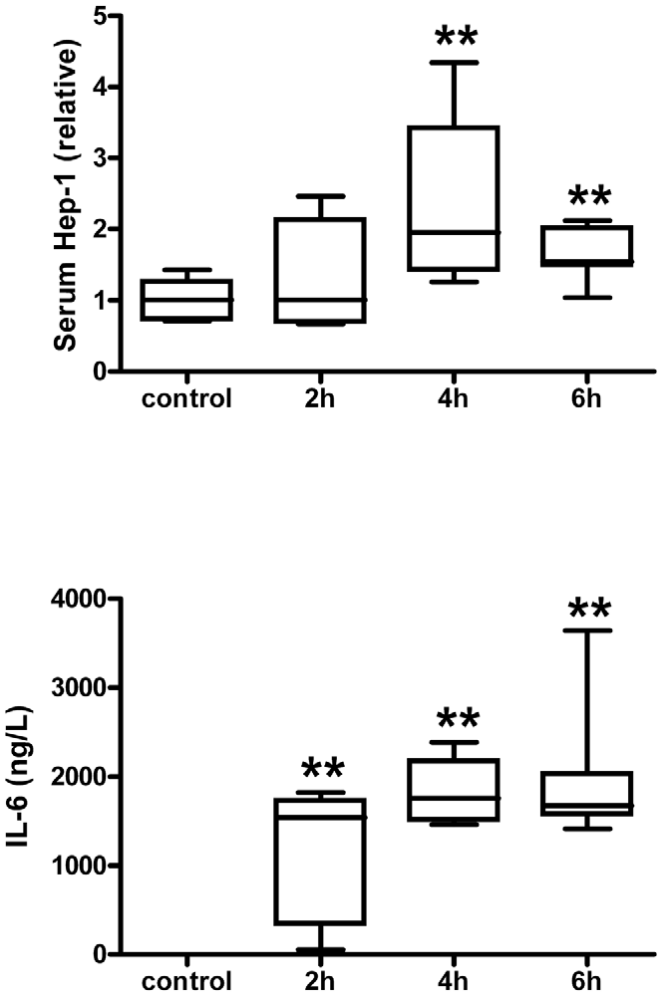
Hep-1 (upper panel) and IL-6 (lower panel) at baseline (control, n = 6), 2 hrs (n = 6), 4 hrs (n = 6) and 6 hrs (n = 7) time course after LPS injection in C57Bl/6 mice strain. IL-6 levels of controls are below the detection limit. Data are depicted as lower quartile, median and upper quartile (boxes), and minimum and maximum ranges (whiskers). Differences of serum hep-1 and IL-6 at 2 h, 4 h and 6 h compared to controls are tested by Mann-Whitney test; **, *P*<0.01, respectively.

To investigate the responses of mouse hepcidin to iron and inflammatory stimuli, intravenous iron and LPS were administered after which hepcidin levels were determined in two separate experiments. In the first experiment, serum Hep-1 and urine Hep-2 were measured 24 hours after iron and LPS stimulus in three different mouse strains. As shown in Figure 6A, serum Hep-1 elevations to both iron and LPS administration were significant in DBA/2 mice and C57Bl/6, but not in BALB/c. Moreover, baseline Hep-2 levels in urine from DBA/2 mice were several magnitudes of units higher than those of the other two mouse strains (Figure 6B). LPS administration had no effect on Hep-2 levels in the urine of C57Bl/6 and BALB/c mice, but significantly decreased urine Hep-2 levels in DBA/2. Iron administration significantly increased Hep-2 levels in C57Bl/6 mice, whereas no effect on Hep-2 levels in urine from BALB/c and DBA/2 mice was observed. It should be mentioned, however, that differences with DBA/2 control mice may have been missed due to restricted urine sample volume which precluded more precise assessment of the Hep-2 levels. To examine the time course of Hep-1 induction upon inflammation in more detail, C57Bl/6 mice were injected with LPS in a second experiment. Serum Hep-1 levels were measured during a 6 hrs time course along with IL-6 levels as a marker for the inflammatory response. As shown in Figure 7, both IL-6 and Hep-1 levels significantly increased in time with a peak expression after about 4 hrs, similar to what has been observed in humans [12]. Altogether these experiments show that Hep-1 and Hep-2 are differently regulated by LPS and iron, whereas this regulation itself is dependent on the genetic background of the mouse strain.

## Discussion

The mouse is a valuable model for understanding human iron disorders. Markedly, mouse studies still report *hepcidin* mRNA levels as a surrogate marker for bioactive hepcidin in its pivotal function to block ferroportin-mediated iron transport. Unlike humans, mice contain two related hepcidin genes of which only *hepcidin-1* seems to be important for iron homeostasis and is primarily expressed in the liver. Instead. the *hepcidin-2* gene is predominantly expressed in the pancreas in some, but not all, mice strains [5], [7]. Here, we developed a methodology to investigate production and fate of both Hep-1 and -2 peptides in various mouse models for iron metabolism disorders.

We found serum Hep-1 levels in C57Bl/6 mice to be increased upon LPS administration in parallel with serum IL-6 levels, which is consistent with a previous report on liver and pancreas *hepcidin-1* expression [7]. Furthermore, we found serum Hep-1 levels to be increased both upon iron and LPS administration in all 3 investigated mice strains (C57Bl/6 and DBA/2 and BALB/c). Similar increases in liver *hepcidin-1* mRNA levels upon both stimuli were previously reported, while pancreas *hepcidin-1* expression only increased upon LPS administration [7], [14]. Thus, our mass spectrometry data show that serum Hep-1 represents an convenient marker in mouse models of inflammation and iron overload. The fact that serum Hep-1 levels correlated with liver *hepcidin-1* expression and disappeared upon acute liver damage by paracetamol intoxication implies that the liver is indeed the foremost source of Hep-1. Mouse serum Hep-1 peptide responses seemed to be modest, especially for LPS compared to Hepcidin-25 responses during human endotoxemia [15]. The latter emphasizes that results from mouse models cannot directly be translated to human iron metabolism.

In contrast to serum Hep-1, the origin of urine Hep-2 remains elusive. We showed that baseline urine Hep-2 levels of DBA/2 mice were substantially higher than that of the other mouse strains and that i.v. iron administration significantly increased urine Hep-2 levels in C57Bl/6 mice, which is in agreement with previous reports on mouse liver *hepcidin-2* expression [7], [14]. Conversely, pancreas *hepcidin-2* expression was previously reported to be most highly expressed in C57Bl/6 mice [7], while i.v. iron administration did not increase mouse pancreas *hepcidin-2* mRNA levels. Furthermore, our current data show that LPS challenge did not raise Hep-2 levels in the urine of C57Bl/6 mice, while these levels in DBA/2 were decreased. Although, the above observations could suggest that the contribution of the liver to urine Hep-2 levels is superior to that of the pancreas, not all observations can be explained by this assumption. First, Krijt *et al*. [7] showed that only pancreatic *hepcidin-2* expression was decreased upon LPS administration in C57Bl/6 and DBA/2 mice. Second, we did not observe a significant increase of urine Hep-2 levels in BALB/c mice, whereas oral iron loading in BALB/c mice was previously shown to result in a clear *hepcidin-2* expression increases in both liver and pancreas [5]. Third, we did not obtain evidence that urine Hep-2 levels were affected by hepatotoxic paracetamol dosage. Accordingly, the latter findings are in-line with the idea that urine Hep-2 does *not* primarily originate from the liver. Thus based on the current state-of-the-art, we tend to believe that neither the pancreas nor the liver is the major source of urine Hep-2. Unfortunately, apart from the liver we did not harvest RNA from other mouse organs to co-investigate *hepcidin-2* expression in our models. These ambiguities on Hep-2 production, however, clearly exemplify that caution should be taken with respect to the extrapolation of results obtained with one mouse strain to another.

A remarkable observation from our study concerned the apparent absence of Hep-1 in urine and Hep-2 in serum. Correspondingly, a recent proteomic analysis of urine from a C57Bl/6 mouse model for glomerulonephritis also yielded the detection of Hep-2, but not Hep-1 [9]. These findings can be explained by the fact that Hep-1, in contrast to human Hepcidin-25, may either not be filtrated by the kidney or is completely degraded and/or reabsorbed by the tubule cells. As Hep-2 was not detectable in serum and seemed unaffected by liver intoxication, it is tempting to speculate that Hep-2 is for a substantial part produced by the kidney or local inflammatory cells and processed into urine. In fact, *hepcidin* expression has been shown in the renal tubules and interstitial inflammatory cells of rats [16] and in kidney biopsies of SLE patients [17]. Nevertheless, local Hep-2 production in the kidney and its potential physiological role requires further investigation. It should be kept in mind, however, that the peptide enrichment procedure in combination with the different characteristics of serum and urine samples may for a substantial part determine whether or not Hep-1 and/or Hep-2 are isolated, implicating that changes in this procedure or altering matrix compositions may cause changes in their relative detection.

Another interesting observation concerned the fact that liver iron, but *not* circulating iron levels, correlated significantly with serum Hep-1 levels, thereby corroborating data on liver iron induced local bone morphogenetic protein 6 (BMP6) levels as the dominant regulator of hepatocyte hepcidin synthesis [18]–[20]. Moreover, serum Hep-1 levels appeared to be an excellent determinant of spleen iron levels, fully consistent with the mode of action of Hep-1, *i.e.* the sequestration of iron in the reticulo-endothelial macrophages by internalisation and subsequent degradation of the sole cellular iron exporter ferroportin [1], [2], [21].

In-line with previous studies showing that defects in the HFE and TfR2 proteins (and their combination) upstream from hepcidin synthesis resulted in low circulating hepcidin levels [22]–[27], our 3 mouse hemochromatosis models all showed impaired Hep-1 production, being most prominently present in double affected Hfe/TfR2^y245x^ mice. This corroborates a concept in which lower levels of bioactive hepcidin result in a clinically more severe hemochromatosis [28], [29]. Accordingly, the combined defect in HFE and TfR2 in man has been shown to lead to more severe iron loading and a juvenile form of hemochromatosis [30], suggesting that these two genes have a least some hepcidin regulatory functions that do not overlap. Furthermore, our findings are in agreement with the reduced serum Hep-1 levels in mice homozygous and heterozygous for targeted deletions in the *hepcidin* and *HFE* genes, as measured by a hepcidin (ferroportin) binding assay [31], [32].

Altogether, our study shows that mass spectrometry is a convenient approach to monitor physiological relevant changes in hepcidin peptide levels in experimental mouse models, which is essential to provide new insights in post-transcriptional regulation, hepcidin function, and kinetics. Moreover, our results indicate that vast inter-species variations in these levels, along with previously described distinct *hepcidin-1* and *-2* expression patterns, may explain the differences in iron homeostasis among mouse strains [33]. Importantly, the current study may facilitate the selection of a proper mouse model to answer a particular research question in iron metabolism.

## Materials and Methods

### Mass spectrometry

Surface Enhanced Laser Desorption Ionization (SELDI) Cu^2+^ affinity-capture TOF MS was used for the routine detection of mouse hepcidin peptides in urine and serum samples as previously described for human samples [12], [13]. Alternatively, mouse hepcidin peptide enrichment was performed by a hydrophobic interaction chromatography bead-based approach followed by peptide profiling on a Matrix-assisted (MA)LDI-TOF MS platform. The identity of Hep-1 in mouse serum was confirmed by high resolution Matrix-Assisted Laser Desorption Ionization - Fourier transform ion cyclotron resonance (MALDI-FTICR) MS and that of Hep-2 by Q-STARXL MS/MS (see Supporting information Materials and Methods S1 for details).

### Experimental mouse models

Mouse models for hemochromatosis (homozygous *Hfe* knock-out mice (Hfe KO), homozygous *Tfr2 ^y245x^* mutated mice (Tfr2^y245x^) and double affected mice (Hfe/TfR2^y245x^), dietary iron challenge, infection (LPS challenge), and acute hepatotoxicity following paracetamol administration were used to validate and evaluate the utility of Hep-1/Hep-2 analysis by mass spectrometry (see Supporting information for details). All animals received human care and study protocols complied with the institutional guidelines. Approval was obtained from the Animal Ethics Committee of the Radboud University Nijmegen (RU-DEC 2008-142), the Austrian Federal Ministry of Science and Research (BMWF-66.011/0111-II/10b/2008 and BMBWK-66.011/0080-BrGT/2005), and the Institutional Animal Care and Use Committee (IACUC) at Saint Louis University School of Medicine (protocol # 1410). Male and female mice were randomly distributed between the study groups.

### Quantification of hepcidin mRNA

Liver *hepcidin-1* mRNA was quantified by real-time RT-PCR (ABI7700) using TaqMan reagents (Applied Biosystems; see Supplemental information for primers and probes). *Hepcidin-1* expression relative to *β-actin* was compared across groups using both the delta Ct method, and the previously described method [34] using REST software (Qiagen).

### Tissue iron concentrations

Liver and spleen non-heme iron concentrations were determined as described [35], and expressed as µg iron per g dry weight.

### Laboratory measurements

Urine creatinine was quantified by enzymatic detection. ALAT levels were determined by NOTOX B.V. (‘s-Hertogenbosch, the Netherlands). Serum IL-6 was determined as described previously [36].

## Supporting Information

Figure S1 Models of the mouse Hep-1 (***A***) and Hep-2 (***B***) protein structures based on the known human hepcidin-25 structure (***C***). For homology modeling, the human hepcidin structure (PDB file 3h0t), was used as input for the YASARA algorithm to swap the human side chains for the corresponding side chains of mouse Hep-1 and -2, respectively, including a standard energy minimization step. This resulting models for mouse Hep-1 and -2 showed the same cysteine-bridge pattern as described for the human hepcidin. Left side: Overview of the structures of the three hepcidin distorted beta –sheets are shown as grey arrow, and the peptide backbone is colored gray. The disulfide bonds are colored yellow, positive residues of arginine (Arg) and lysine (Lys) are picture in blue, the negative residue of asparctic acid (Asp) in red. Right side: the molecule displayed with solvent accessible surface. The molecule is colored gray, except for the side-chains of positive (blue) and negative (red) residues. Mouse Hep-1; sequence: DTNFPICIFCCKCCNNSQCGICCKT. Mass: 2754 Da; pI: 7.7. Mouse Hep-2; sequence: DINFPICRFCCQCCNKPSCGICCEE. Mass: 2821 Da; pI: 4.9 Human hepcidin; sequence: DTHFPICIFCCGCCHRSKCGMCCKT. Mass: 2789 Da; pI: 8.2.
(PDF)

Figure S2 Hep-1 contains 4 disulphide bridges. TOF MS profile of a FVB mouse serum sample before (***A***) and after reduction with DTT (***B***) and alkylation with IAA (***C***). Respective mass shifts of +8 Da and +456 Da specify the reduction of 4 disulfide bonds. Note that a partial reaction resulted in an alkylation ladder (peaks indicted with *) with size difference of 57 Da per moiety (from 5 to 8 modifications). Intensity is given in arbitrary units (AU).
(PDF)

Figure S3 Hep-2 contains 4 disulphide bridges. Hep-2 was enriched by IMAC-Cu^2+^ on-chip chemistry from urine of a C57Bl6 mouse and analyzed by Q-STARXL MS before (***A***) and after (***B***) reduction with DDT; upper spectrum shows reduced Hep-2 (m/z 2828) and lower spectrum: native Hep-2 (m/z 2821). Note that monoisotopic [M+H]^+^ masses as measured by Q-STARXL MS are about 1 Dalton lighter than the average masses measured by TOF MS.
(PDF)

Figure S4 Hep-2 identification by Q-STARXL MS/MS. Hep-2 was enriched by IMAC-Cu2+ on-chip chemistry from urine of a C57Bl6 mouse after which Q-STARXL MS/MS was used to generate fragmentation spectra of the reduced m/z 2828 peptide (***A***). Database search results for the MS/MS analysis of the m/z 2828 peptide are shown in panel ***B***.
(PDF)

Figure S5 Identification of Hep-1 by MALDI-FTICR MS. Serum Hep-1 from FVB mouse was enriched by IMAC-Cu^2+^ on-chip chemistry after which MALDI-FTICR MS was used for an accurate mass measurement. The upper panel shows a part of the resulting MALDI-FTICR spectrum. The peak of interest was measured at m/z 2753.032 using external calibration. The mass difference with the signal at m/z 2637.989 exactly corresponds to an aspartic acid residue, which is only in agreement with a single amino acid truncation at the N-terminus of Hep-1 (Table S1). Additional evidence for the fact that the peptides at 2753 and 2637 have the same amino acid composition is found in their similar isotopic distributions, shown in the enlarged parts of the lower panel. In contrast, the peaks at m/z 2908 and 2791 also seem to be related, but their mass difference of 117 does not correspond to any amino acid residue. The exact mass difference between m/z 2908 and 2753 is 155.5, implying two different amino acid compositions. Another indication that these two peptides are two distinct structures is the different isotopic distributions of 2753 and 2908. From similar reasoning it was concluded that the signal at m/z 2673 is not related to any of the four peptides in the MALDI-FTICR spectrum.
(PDF)

Figure S6 Distinct urine and serum Hep-1 and Hep-2 forms in diverse mouse strains. ***A*** **,** TOF MS serum profile of a FVB mouse, ***B*** **,** serum profile of a DBA/2 mouse, Da. ***C*** **,** urine profile of FVB mouse, ***D*** **,** urine profile of DBA/2 mouse. Note that DBA/2 mice have, in contrast to other mouse strains, an Asn16Lys substitution in mature Hep-1 resulting in a theoretical mass of 2768 Da and a Ser18 Phe substitution; in mature Hep-2 resulting in a theoretical mass of 2881 Da (see Figure S1). The measured mass shifts in Hep-1/-2 in serum and urine from DBA/2, match with the altered amino acid composition. Positions of Hep-1 and Hep-2 are indicated. Peak intensity is given in arbitrary units (AU).
(PDF)

Figure S7 TOF MS profiles showing isoforms of Hep-2 in the urine of ***A***, a DBA/2 mouse (n = 1), and ***B*** **,** C57Bl/6 mice (n = 3). Note that Hep-2 from DBA/2 mice has a deviant mass of 2881 Da (instead of 2821 Da for other mouse strains) due to a Ser18 Phe substitution in the mature part of Hep-2. The masses of the indicated Hep-2 isoforms match to the theoretical masses of amino-terminal truncated 23, 22 and 20 amino acid (aa) forms of Hep-2. Panel B illustrates the inter-mouse heterogeneity in Hep-2 (isoform) levels in mouse urine. Intensity is given in arbitrary units (AU).
(PDF)

Table S1 (PDF)

Materials and Methods S1 (PDF)
