# Supplementary material for: Mass Spectrometry Analysis of Hepcidin Peptides in Experimental Mouse Models
Source: PLoS One. 2011 Mar 8;6(3):e16762. doi: 10.1371/journal.pone.0016762 (PMC3050808; doi:10.1371/journal.pone.0016762)
Supplement: Figure S1 — Models of the mouse Hep-1 (A) and Hep-2 (B) protein structures based on the known human hepcidin-25 structure (C). For homology modeling, the human hepcidin structure (PDB file 3h0t), was used as input for the YASARA algorithm to swap the human side chains for the corresponding side chains of mouse Hep-1 and -2, respectively, including a standard energy minimization step. This resulting models for mouse Hep-1 and -2 showed the same cysteine-bridge pattern as described for the human hepcidin. Left side: Overview of the structures of the three hepcidin distorted beta –sheets are shown as grey arrow, and the peptide backbone is colored gray. The disulfide bonds are colored yellow, positive residues of arginine (Arg) and lysine (Lys) are picture in blue, the negative residue of asparctic acid (Asp) in red. Right side: the molecule displayed with solvent accessible surface. The molecule is colored gray, except for the side-chains of positive (blue) and negative (red) residues. Mouse Hep-1; sequence: DTNFPICIFCCKCCNNSQCGICCKT. Mass: 2754 Da; pI: 7.7. Mouse Hep-2; sequence: DINFPICRFCCQCCNKPSCGICCEE. Mass: 2821 Da; pI: 4.9 Human hepcidin; sequence: DTHFPICIFCCGCCHRSKCGMCCKT. Mass: 2789 Da; pI: 8.2. (PDF) [file pone.0016762.s001.pdf]

Supporting Figure S1

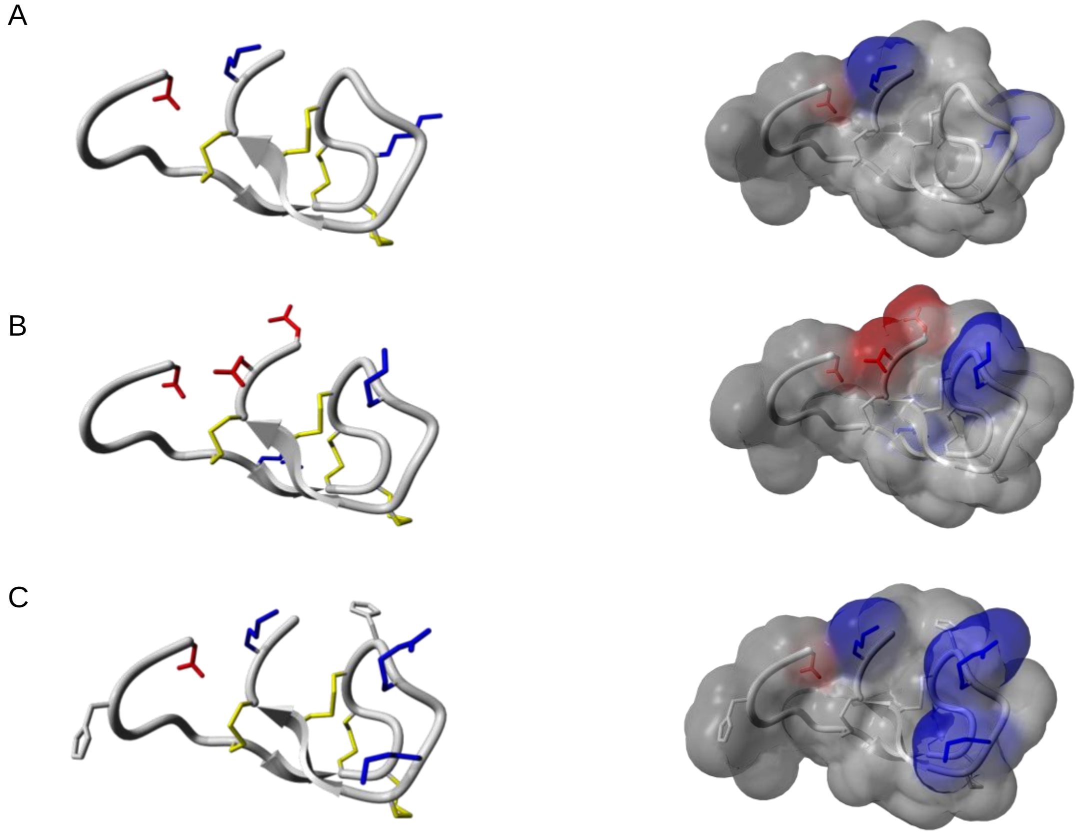

Figure S1. Models of the mouse Hep-1 (***A***) and Hep-2 (***B***) protein structures based on the known human hepcidin-25 structure (***C***). For homology modeling, the human hepcidin structure (PDB file 3h0t; Jordan *et al*. [1]), was used as input for the YASARA algorithm [2] to swap the human side chains for the corresponding side chains of mouse Hep-1 and -2, respectively, including a standard energy minimization step. This resulting models for mouse Hep-1 and -2 showed the same cysteine-bridge pattern as described for the human hepcidin. *Left side:* Overview of the structures of the three hepcidin distorted beta –sheets are shown as grey arrow, and the peptide backbone is colored gray. The disulfide bonds are colored yellow, positive residues of arginine (Arg) and lysine (Lys) are picture in blue, the negative residue of asparctic acid (Asp) in red. *Right side:* the molecule displayed with solvent accessible surface. The molecule is colored gray, except for the side-chains of positive (blue) and negative (red) residues. Mouse Hep-1; sequence: DTNFPICIFCCKCCNNSQCGICCKT. Mass: 2754 Da; pI: 7.7. Mouse Hep-2; sequence: DINFPICRFCCQCCNKPSCGICCEE. Mass: 2821 Da; pI: 4.9 Human hepcidin; sequence: DTHFPICIFCCGCCHRSKCGMCCKT. Mass: 2789 Da; pI: 8.2.

[1] Jordan JB, Poppe L, Haniu M, Arvedson T, Syed R, Li V, et al. (2009) Hepcidin revisited, disulfide connectivity, dynamics, and structure. J Biol Chem 284: 24155-24167.
[2] Krieger E, Koraimann G, Vriend G (2002) Increasing the precision of comparative models with YASARA NOVA--a self-parameterizing force field. Proteins 47: 393-402.
