# Supplementary material for: Mass Spectrometry Analysis of Hepcidin Peptides in Experimental Mouse Models
Source: PLoS One. 2011 Mar 8;6(3):e16762. doi: 10.1371/journal.pone.0016762 (PMC3050808; doi:10.1371/journal.pone.0016762)
Supplement: Figure S2 — Hep-1 contains 4 disulphide bridges. TOF MS profile of a FVB mouse serum sample before (A) and after reduction with DTT (B) and alkylation with IAA (C). Respective mass shifts of +8 Da and +456 Da specify the reduction of 4 disulfide bonds. Note that a partial reaction resulted in an alkylation ladder (peaks indicted with *) with size difference of 57 Da per moiety (from 5 to 8 modifications). Intensity is given in arbitrary units (AU). (PDF) [file pone.0016762.s002.pdf]

Supporting Figure S2

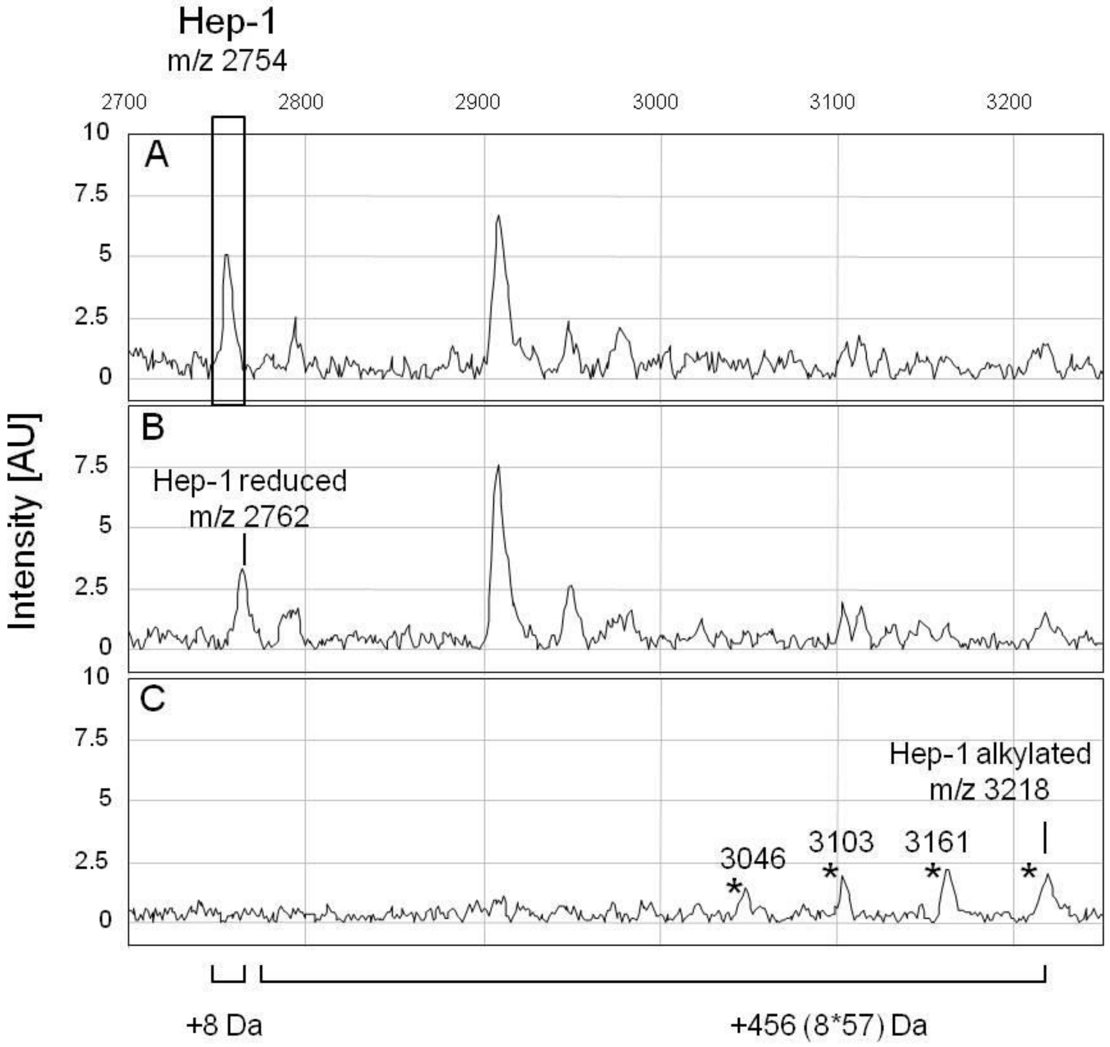

Figure S2. Hep-1 contains 4 disulphide bridges. TOF MS profile of a FVB mouse serum sample before (**A**) and after reduction with DTT (**B**) and alkylation with IAA (**C**). Respective mass shifts of +8 Da and +456 Da specify the reduction of 4 disulfide bonds. Note that a partial reaction resulted in an alkylation ladder (peaks indicted with *) with size difference of 57 Da per moiety (from 5 to 8 modifications). Intensity is given in arbitrary units (AU).
