# Supplementary material for: Mass Spectrometry Analysis of Hepcidin Peptides in Experimental Mouse Models
Source: PLoS One. 2011 Mar 8;6(3):e16762. doi: 10.1371/journal.pone.0016762 (PMC3050808; doi:10.1371/journal.pone.0016762)
Supplement: Figure S3 — Hep-2 contains 4 disulphide bridges. Hep-2 was enriched by IMAC-Cu2+ on-chip chemistry from urine of a C57Bl6 mouse and analyzed by Q-STARXL MS before (A) and after (B) reduction with DDT; upper spectrum shows reduced Hep-2 (m/z 2828) and lower spectrum: native Hep-2 (m/z 2821). Note that monoisotopic [M+H]+ masses as measured by Q-STARXL MS are about 1 Dalton lighter than the average masses measured by TOF MS. (PDF) [file pone.0016762.s003.pdf]

Supporting Figure S3

A

B

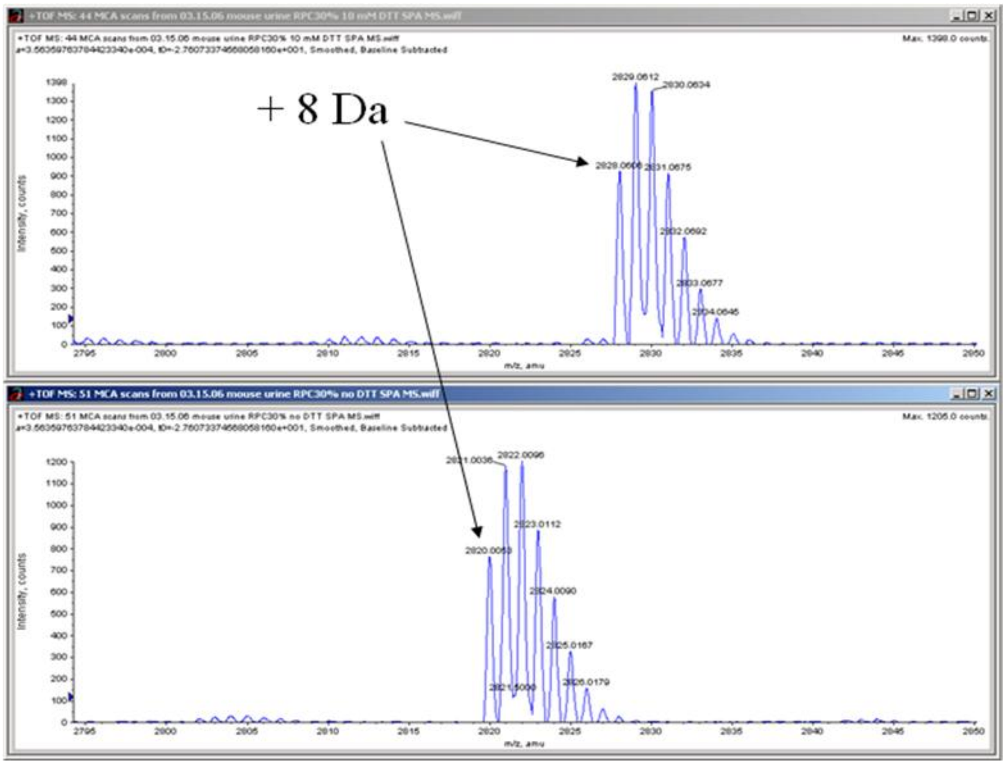

Figure S3. Hep-2 contains 4 disulphide bridges. Hep-2 was enriched by IMAC-Cu^{2+} on-chip chemistry from urine of a C57Bl6 mouse and analyzed by Q-STARXL MS before (**A**) and after (**B**) reduction with DDT; upper spectrum shows reduced Hep-2 (m/z 2828) and lower spectrum: native Hep-2 (m/z 2821). Note that monoisotopic $[M+H]^+$ masses as measured by Q-STARXL MS are about 1 Dalton lighter than the average masses measured by TOF MS.
