# Supplementary material for: Mass Spectrometry Analysis of Hepcidin Peptides in Experimental Mouse Models
Source: PLoS One. 2011 Mar 8;6(3):e16762. doi: 10.1371/journal.pone.0016762 (PMC3050808; doi:10.1371/journal.pone.0016762)
Supplement: Figure S4 — Hep-2 identification by Q-STARXL MS/MS. Hep-2 was enriched by IMAC-Cu2+ on-chip chemistry from urine of a C57Bl6 mouse after which Q-STARXL MS/MS was used to generate fragmentation spectra of the reduced m/z 2828 peptide (A). Database search results for the MS/MS analysis of the m/z 2828 peptide are shown in panel B. (PDF) [file pone.0016762.s004.pdf]

Supporting Figure S4

A

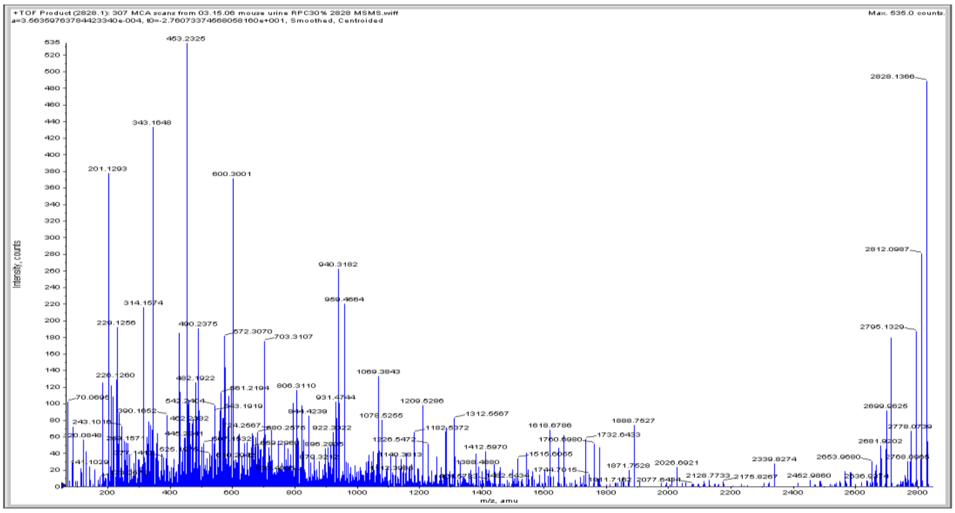

B

Ions score is -10*Log(P), where P is the probability that the observed match is a random event.
Individual ions scores > 24 indicate identity or extensive homology (p<0.05).
Protein scores are derived from ions scores as a non-probabilistic basis for ranking protein hits.

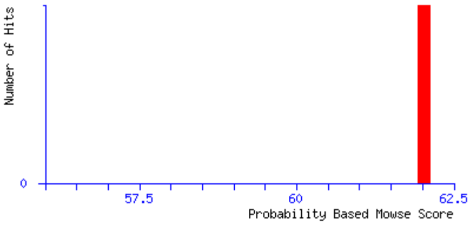

1. Q80T19 **Mass:** 9404 **Score:** 62 **Queries matched:** 1
(HEPC2_MOUSE) Hepcidin-2 precursor Hepcidin-2 precursor
☐ Check to include this hit in error tolerant search

	Query	Observed	Mr(expt)	Mr(calc)	Delta	Miss	Score	Exp[illegible]
☑	1	2828.1000	2827.0927	2827.0847	0.0081	0	62	8.3e[illegible]

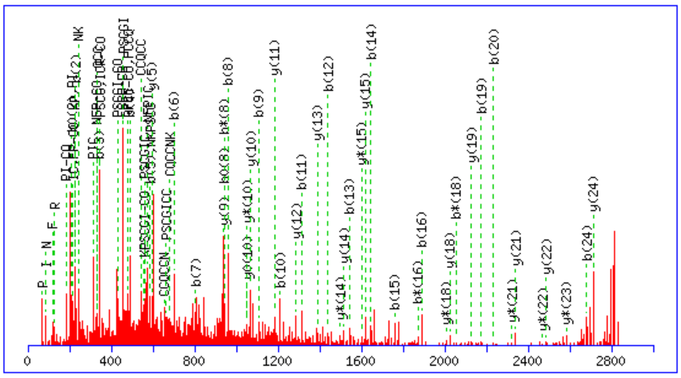

#	Immon.	b	b*	b⁰	Seq.	y	y*	y⁰	#
1	88.0393	116.0342		98.0237	D				25
2	86.0964	229.1183		211.1077	I	2713.0650	2696.0384	2695.0544	24
3	87.0553	343.1612	326.1347	325.1506	N	2599.9809	2582.9544	2581.9704	23
4	120.0808	490.2296	473.2031	472.2191	F	2485.9380	2468.9114	2467.9274	22
5	70.0651	587.2824	570.2558	569.2718	P	2338.8696	2321.8430	2320.8590	21
6	86.0964	700.3664	683.3399	682.3559	I	2241.8168	2224.7903	2223.8063	20
7	76.0215	803.3756	786.3491	785.3651	C	2128.7328	2111.7062	2110.7222	19
8	129.1135	959.4767	942.4502	941.4662	R	2025.7236	2008.6970	2007.7130	18
9	120.0808	1106.5451	1089.5186	1088.5346	F	1869.6225	1852.5959	1851.6119	17
10	76.0215	1209.5543	1192.5278	1191.5438	C	1722.5541	1705.5275	1704.5435	16
11	76.0215	1312.5635	1295.5369	1294.5529	C	1619.5449	1602.5183	1601.5343	15
12	101.0709	1440.6221	1423.5955	1422.6115	Q	1516.5357	1499.5092	1498.5251	14
13	76.0215	1543.6313	1526.6047	1525.6207	C	1388.4771	1371.4506	1370.4666	13
14	76.0215	1646.6404	1629.6139	1628.6299	C	1285.4679	1268.4414	1267.4574	12
15	87.0553	1760.6834	1743.6568	1742.6728	N	1182.4588	1165.4322	1164.4482	11
16	101.1073	1888.7783	1871.7518	1870.7678	K	1068.4158	1051.3893	1050.4053	10
17	70.0651	1985.8311	1968.8045	1967.8205	P	940.3209		922.3103	9
18	60.0444	2072.8631	2055.8366	2054.8526	S	843.2681		825.2575	8
19	76.0215	2175.8723	2158.8457	2157.8617	C	756.2361		738.2255	7
20	30.0338	2232.8938	2215.8672	2214.8832	G	653.2269		635.2163	6
21	86.0964	2345.9778	2328.9513	2327.9673	I	596.2054		578.1949	5
22	76.0215	2448.9870	2431.9604	2430.9764	C	483.1214		465.1108	4
23	76.0215	2551.9962	2534.9696	2533.9856	C	380.1122		362.1016	3
24	102.0550	2681.0388	2664.0122	2663.0282	E	277.1030		259.0925	2
25	102.0550				E	148.0604		130.0499	1

Figure S4. Hep-2 identification by Q-STARXL MS/MS. Hep-2 was enriched by IMAC-Cu^{2+} on-chip chemistry from urine of a C57Bl6 mouse after which Q-STARXL MS/MS was used to generate fragmentation spectra of the reduced m/z 2828 peptide (**A**).Database search results for the MS/MS analysis of the m/z 2828 peptide are shown in panel **B**.
