# Supplementary material for: Mass Spectrometry Analysis of Hepcidin Peptides in Experimental Mouse Models
Source: PLoS One. 2011 Mar 8;6(3):e16762. doi: 10.1371/journal.pone.0016762 (PMC3050808; doi:10.1371/journal.pone.0016762)
Supplement: Figure S5 — Identification of Hep-1 by MALDI-FTICR MS. Serum Hep-1 from FVB mouse was enriched by IMAC-Cu2+ on-chip chemistry after which MALDI-FTICR MS was used for an accurate mass measurement. The upper panel shows a part of the resulting MALDI-FTICR spectrum. The peak of interest was measured at m/z 2753.032 using external calibration. The mass difference with the signal at m/z 2637.989 exactly corresponds to an aspartic acid residue, which is only in agreement with a single amino acid truncation at the N-terminus of Hep-1 (Table S1). Additional evidence for the fact that the peptides at 2753 and 2637 have the same amino acid composition is found in their similar isotopic distributions, shown in the enlarged parts of the lower panel. In contrast, the peaks at m/z 2908 and 2791 also seem to be related, but their mass difference of 117 does not correspond to any amino acid residue. The exact mass difference between m/z 2908 and 2753 is 155.5, implying two different amino acid compositions. Another indication that these two peptides are two distinct structures is the different isotopic distributions of 2753 and 2908. From similar reasoning it was concluded that the signal at m/z 2673 is not related to any of the four peptides in the MALDI-FTICR spectrum. (PDF) [file pone.0016762.s005.pdf]

Supporting Figure S5

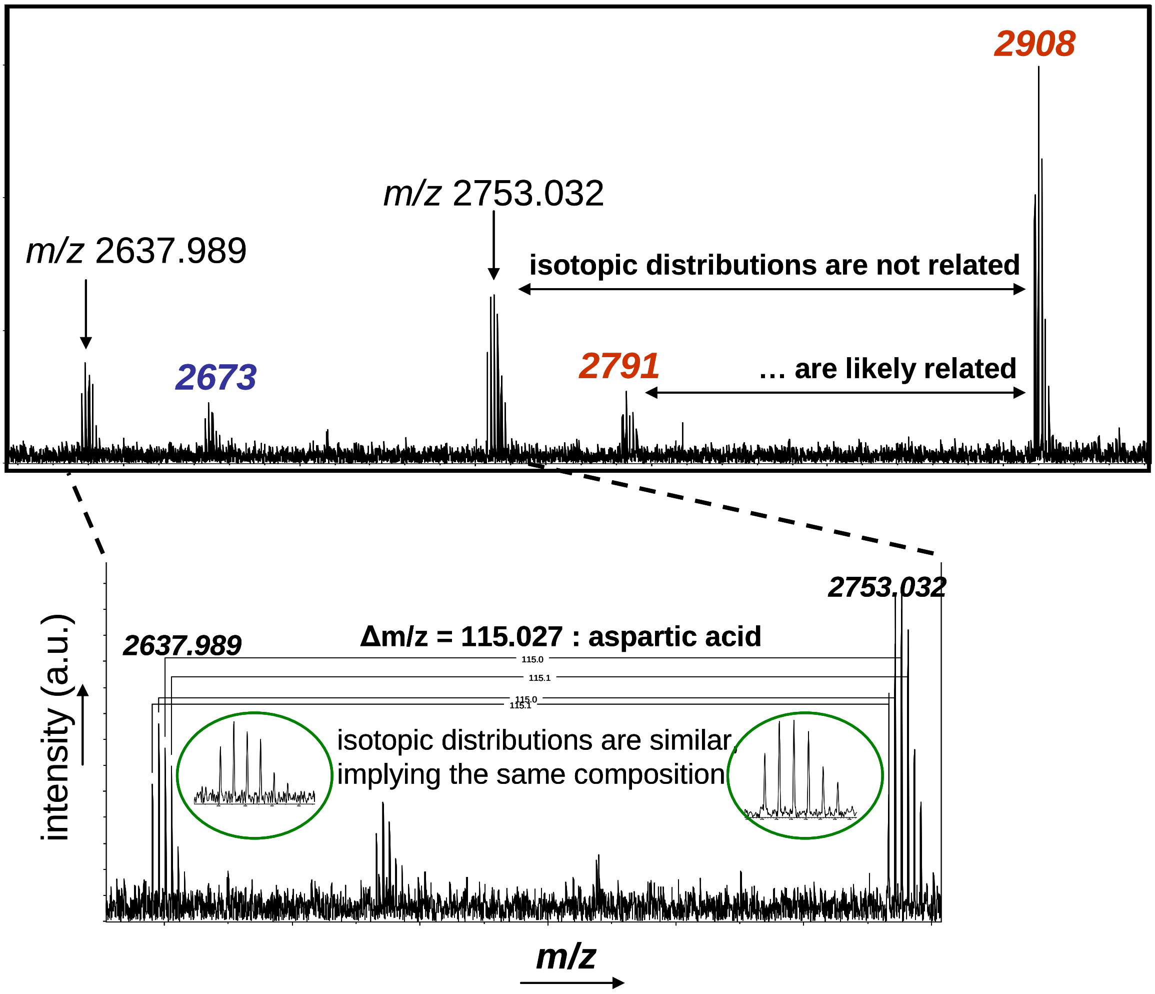

Figure S5. Identification of Hep-1 by MALDI-FTICR MS. Serum Hep-1 from FVB mouse was enriched by IMAC-Cu^{2+} on-chip chemistry after which MALDI-FTICR MS was used for an accurate mass measurement. The upper panel shows a part of the resulting MALDI-FTICR spectrum. The peak of interest was measured at *m/z* 2753.032 using external calibration. The mass difference with the signal at *m/z* 2637.989 exactly corresponds to an aspartic acid residue, which is only in agreement with a single amino acid truncation at the N-terminus of Hep-1 (Table S1). Additional evidence for the fact that the peptides at 2753 and 2637 have the same amino acid composition is found in their similar isotopic distributions, shown in the enlarged parts of the lower panel. In contrast, the peaks at m/z 2908 and 2791 also seem to be related, but their mass difference of 117 does not correspond to any amino acid residue. The exact mass difference between m/z 2908 and 2753 is 155.5, implying two different amino acid compositions. Another indication that these two peptides are two distinct structures is the different isotopic distributions of 2753 and 2908. From similar reasoning it was concluded that the signal at *m/z* 2673 is not related to any of the four peptides in the MALDI-FTICR spectrum.
