# Supplementary material for: Mass Spectrometry Analysis of Hepcidin Peptides in Experimental Mouse Models
Source: PLoS One. 2011 Mar 8;6(3):e16762. doi: 10.1371/journal.pone.0016762 (PMC3050808; doi:10.1371/journal.pone.0016762)
Supplement: Figure S6 — Distinct urine and serum Hep-1 and Hep-2 forms in diverse mouse strains. A , TOF MS serum profile of a FVB mouse, B , serum profile of a DBA/2 mouse, Da. C , urine profile of FVB mouse, D , urine profile of DBA/2 mouse. Note that DBA/2 mice have, in contrast to other mouse strains, an Asn16Lys substitution in mature Hep-1 resulting in a theoretical mass of 2768 Da and a Ser18 Phe substitution; in mature Hep-2 resulting in a theoretical mass of 2881 Da (see Figure S1). The measured mass shifts in Hep-1/-2 in serum and urine from DBA/2, match with the altered amino acid composition. Positions of Hep-1 and Hep-2 are indicated. Peak intensity is given in arbitrary units (AU). (PDF) [file pone.0016762.s006.pdf]

Supporting Figure S6

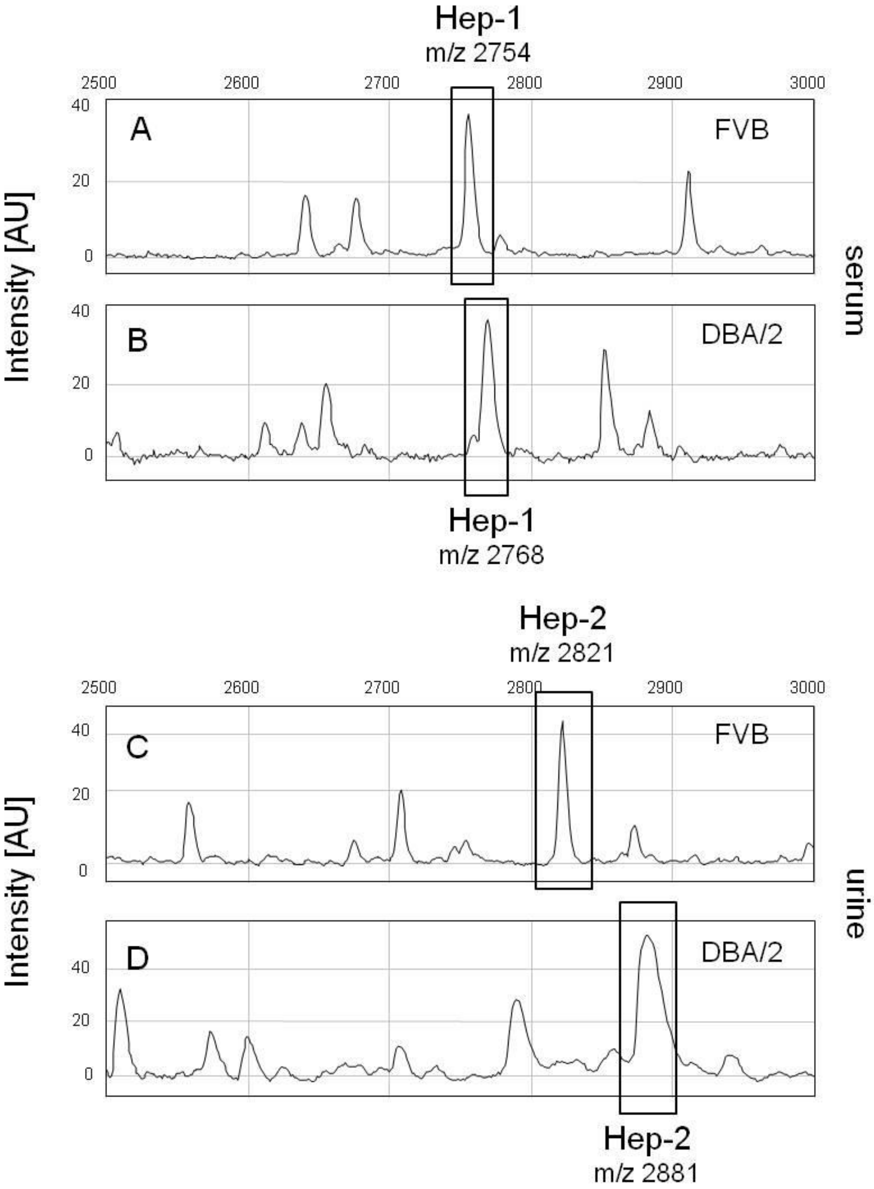

Figure S6. Distinct urine and serum Hep-1 and Hep-2 forms in diverse mouse strains. ***A*,** IMAC-Cu^{2+} TOF MS serum profile of a FVB mouse, ***B*,** serum profile of a DBA/2 mouse, Da. ***C*,** urine profile of FVB mouse, ***D*,** urine profile of DBA/2 mouse. Note that DBA/2 mice have, in contrast to other mouse strains, an Asn16Lys substitution in mature Hep-1 resulting in a theoretical mass of 2768 Da and a Ser18 Phe substitution; in mature Hep-2 resulting in a theoretical mass of 2881 Da (see Figure S1). The measured mass shifts in Hep-1/-2 in serum and urine from DBA/2, match with the altered amino acid composition. Positions of Hep-1 and Hep-2 are indicated. Peak intensity is given in arbitrary units (AU).
