# Supplementary material for: Mass Spectrometry Analysis of Hepcidin Peptides in Experimental Mouse Models
Source: PLoS One. 2011 Mar 8;6(3):e16762. doi: 10.1371/journal.pone.0016762 (PMC3050808; doi:10.1371/journal.pone.0016762)
Supplement: Figure S7 — TOF MS profiles showing isoforms of Hep-2 in the urine of A, a DBA/2 mouse (n = 1), and B , C57Bl/6 mice (n = 3). Note that Hep-2 from DBA/2 mice has a deviant mass of 2881 Da (instead of 2821 Da for other mouse strains) due to a Ser18 Phe substitution in the mature part of Hep-2. The masses of the indicated Hep-2 isoforms match to the theoretical masses of amino-terminal truncated 23, 22 and 20 amino acid (aa) forms of Hep-2. Panel B illustrates the inter-mouse heterogeneity in Hep-2 (isoform) levels in mouse urine. Intensity is given in arbitrary units (AU). (PDF) [file pone.0016762.s007.pdf]

Supporting Figure S7

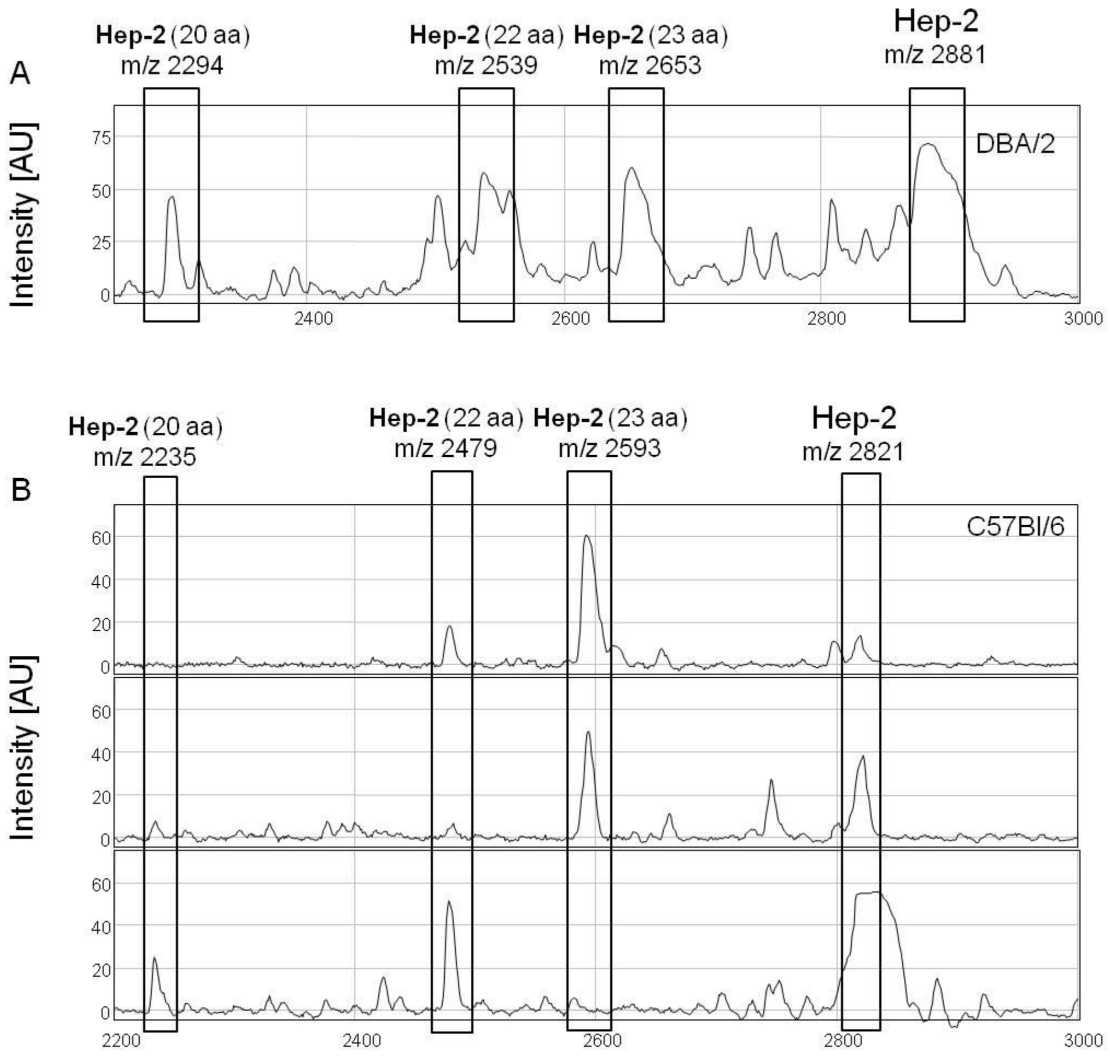

Figure S7. IMAC-Cu^{2+} TOF MS profiles showing isoforms of Hep-2 in the urine of **A,** a DBA/2 mouse (n=1), and **B,** C57Bl/6 mice (n=3). Note that Hep-2 from DBA/2 mice has a deviant mass of 2881 Da (instead of 2821 Da for other mouse strains) due to a Ser18 Phe substitution in the mature part of Hep-2. The masses of the indicated Hep-2 isoforms match to the theoretical masses of amino-terminal truncated 23, 22 and 20 amino acid (aa) forms of Hep-2. Panel B illustrates the inter-mouse heterogeneity in Hep-2 (isoform) levels in mouse urine. Intensity is given in arbitrary units (AU).
