# Supplementary material for: Mass Spectrometry Analysis of Hepcidin Peptides in Experimental Mouse Models
Source: PLoS One. 2011 Mar 8;6(3):e16762. doi: 10.1371/journal.pone.0016762 (PMC3050808; doi:10.1371/journal.pone.0016762)
Supplement: Table S1 — (PDF) [file pone.0016762.s008.pdf]

Supporting Table S1

Candidate 8*Cys mouse peptides with $[M+H]^+$ monoisotopic masses of m/z 2753.028 ± 50 ppm.

#ID	Protein name	Amino acids	Peptide sequence	theoretical m/z[1]	Δ Mass	ΔPPM
ENSMUSG00000050440	Hepcidin-1	58-83	DTNFPICIFCCKCCNNSQCGICCKT	2753.0236	0	0
ENSMUSG00000031765	Metallothionein-1	94-122	LTSSSSFLGCCSCCPVGCSKCAQGCVCK	2753.0447	-0.021	-7.68
ENSMUSG00000069583	Keratin associated protein 12-1	29-56	CQASCFVSSPCQPSCCVSSSCQSACCR	2752.9468	0.077	27.9
ENSMUSG00000031802	Per-hexamer repeat gene 4	2-25	CIYVCGVCLCVCFSVCMCVHVLCVY	2753.1078	-0.084	-30.6
ENSMUSG00000063251	Keratin associated protein 4-1	6-27	GSVCSEESCGQGCCQPSCCQTTCCRTT	2752.8951	0.128	46.7
ENSMUSG00000049593	Late cornified envelope 1H	35-67	KCPPVSSCCSLGSGGCCGSSSGGCCGSSSGGC	2752.8951	0.128	46.7

Note that monoisotopic [+H] masses as measured by MALDI-FTICR are about 1 Dalton lower than the average mass measured by TOF MS. [1]Theoretical monoisotopic m/z values were calculated as a singly protonated precursor ion from a peptide that contains 4 disulfide bridges using the ChemCalc algorithm (www.chemcalc.org).
