# Supplementary material for: Mass Spectrometry Analysis of Hepcidin Peptides in Experimental Mouse Models
Source: PLoS One. 2011 Mar 8;6(3):e16762. doi: 10.1371/journal.pone.0016762 (PMC3050808; doi:10.1371/journal.pone.0016762)
Supplement: Materials and Methods S1 — (PDF) [file pone.0016762.s009.pdf]

Supporting Material & Methods

Time-of-flight Mass Spectrometry (TOF MS) on mouse serum and urine samples

In the current study, a 5 µl serum/urine sample spiked with 10 nM internal standard, was routinely applied to Cu^{2+}-loaded Immobilized Metal Affinity Capture 30 (IMAC30; Bio-Rad) protein chip arrays, all equilibrated with appropriate buffers. Unless stated otherwise, mouse hepcidin levels were normalised to an internal hepcidin-24 standard and expressed relative to the mean of the baseline value. Synthetic mouse Hep-1 (Peptides International) was used for mass tracing. Alternatively, mouse hepcidin peptides were enriched from 10 µl serum/urine using MB-HIC C8 beads (Bruker Daltonics), after which peptide spectra were generated on a Microflex LT MALDI-TOF MS platform (Bruker Daltonics). In the latter case bombesin (m/z 1620; Bachem) was used as an internal standard for quality control.

Serum Hep-1 identification.

The identity of Hep-1 in serum from FVB mouse was confirmed by high resolution Matrix-Assisted Laser Desorption Ionization - Fourier transform ion cyclotron resonance (MALDI-FTICR) MS carried out on a Bruker apex-ultra 9.4 tesla FTICR mass spectrometer. This instrument was equipped with a dual source (Apollo II), two ion funnels, a selection quadrupole (Q) in the front-end, and an Infinity™ ICR cell (Bruker Daltonics). The system was (externally) calibrated with a commercially available peptide mixture spotted on an 600/384 AnchorChip (Bruker Daltonics) with 4-hydroxy-alpha-cyanocinnamic acid as a matrix. The Hep-1 sample on a SELDI protein chip array was mounted in the MALDI-source using a custom-made adapter plate (Bruker Daltonics). A SmartBeam™ 200 Hz solid-state laser, operated a frequency of 100 Hz, was used for ionization. The irradiation spot size was approximately of 200 µm. The ions generated from 50 laser shots were accumulated in the hexapole and then transferred through the RF-only quadrupole to the collision cell. This process was repeated eight times, on different raster spots, per sample. The 450 laser shots accumulated in the collision cell were then transferred to the ICR cell for mass analysis. The

quadrupole was set to an m/z of 400 with a selection window of 10 Da and RF amplitude of 3000 Vpp. Each spectrum was generated by accumulation of 16 scans with 1M data points. All data were acquired using the Bruker apexControl software and evaluated using Bruker DataAnalysis software. To identify from which mouse protein a peptide at *m/z* 2753 could potentially be derived, the mass for all possible (internal) substrings containing eight cysteines (*i.e.* to yield 4 intramolecular S-S bridges) in each of the 45,430 proteins of the complete mouse proteome (downloaded from BioMart on November 4th, 2009) was determined by a custom PERL script (written by BED) based on the masses of all amino acid (aa) therein plus water. The theoretical $[M+H]^+$ monoisotopic masses of these peptides were calculated by the ChemCalc algorithm (www.chemcalc.org) assuming that all 8 cysteine residues were involved in the 4 disulphide bridges typical for hepcidin peptides. Note that due to the high resolution of the FTICR mass analyzer, the monoisotopic mass $[M+H]^+$ is measured in contrast to the average mass that is measured by TOF MS.

Urine Hep-2 identification.

Hepcidin containing urine samples of C57Bl/6 mouse were air-dried on a SELDI protein chip array after which proteins were on-chip reduced by addition of 10 mM dithiothreitol (DTT) and incubation at 70 °C as described previously [1]. Single MS spectra for unreduced and DTT-reduced samples were generated by Q-STARXL mass spectrometer (Applied Biosystems) equipped with a Ciphergen PCI-1000 ProteinChip Interface. Reduced samples were used to acquire tandem mass spectrometry (MS/MS) spectra by collision-induced dissociation (CID). Data were analyzed by the database-mining tool Mascot (Matrix Science). Similar to FTICR MS, Q-STAR analysis determines the monoisotopic mass $[M+H]^+$. In principle, MALDI-FTICR MS (as used for Hep-1 identification) would also have been appropriate for Hep-2 identification, but for logistic reasons it was more convenient to use Q-STARXL MS in this case. Notably, the Hep-2 levels in urine from DBA mice is generally higher than that of C57Bl/6 mice, but due to its increased availability urine from the latter strain was used for identification purposes.

Experimental mouse models

1. Hemochromatosis models. Male Hfe knockout mice [2] and transferrin receptor 2^{Y245X} mice [3] were bred on an FVB background for more than 7 generations. These two mice strains were crossed with each other and bred to homozygosis for each mutant allele. Colonies were maintained as homozygotes for each allele individually, and as compound mutant homozygotes. Mice were fed standard chow with 270 mg/kg iron after weaning at 21 days. At 5 weeks of age, the mice were killed by exposure to hypercarbia followed by exsanguination, and tissues harvested.

2. Iron and LPS challenge. These studies comprise two parts, which were both performed in male mice at 12 weeks of age, fed on a standard rodent chow with 174 mg/kg iron. Serum is collected by retrobulbar puncture. In the *first* study, we determined serum Hep-1 and urine Hep-2 levels in samples obtained from 3 different mouse strains (C57Bl/6; DBA/2 and BALB/c), at baseline and 24 hrs after iron sucrose (Fe^{3+} 100 mg/kg i.p. (Venofer), bacterial lipopolysaccharide (LPS) administration (1 mg/kg i.p., from *Escherichia coli* 055:B5, Sigma L4005) or solvent (PBS) treatment. 24 Hrs after LPS, iron and PBS administration, mice were housed in metabolic cages for 12 hrs with free access to water but no chow (between 6 p.m. and 6 a.m.) to collect urine. Serum was prepared from blood collected when removing the mice from the metabolic cages.

The *second* study was a time-course experiment, in which serum of C57Bl/6 mice was collected at 6 a.m; 2, 4 and 6 hrs after LPS 1 mg/kg i.p. Controls received no injection.

3. Acute hepatotoxicity following paracetamol administration. For these studies 8 weeks old male FVB mice were provided with standard chow containing 178 mg/kg iron. Mice were administered a single intraperitoneal dose of 350 mg/kg body weight paracetamol (Sigma-Aldrich). 24 Hrs after administration urine was collected using metabolic cages and blood samples were obtained by retrobulobar punction. Notably, this dosage of paracetamol was

not nephrotoxic in that no microscopic damage was observed in the kidney tissue and urine markers for nephrotoxicity (NGAL and Kim-1) did not differ from control mice.

Quantification of hepcidin mRNA

Primers and probes (Alpha Innotech) used for RT-PCR were: Hepcidin-1 forward: CCTATCTCCATCAACAGGTG, reverse: AACAGATACCACACTGGGAA, and probe: 6FAM-CCCTGCTTTCTTCCCCGTGCAAAGT-TAMRA. β-actin forward: CCGTGAAAAGATGACC CAGATCATG, reverse: TCTTCATGAGGTAGTCCGTCAGGTC and probe: 6FAM-TACG AGGGCTATGCTCTCCCTCACGCT-TAMRA. Verification of RNA integrity of each sample after DNAse treatment was performed by analysis of ethidium bromide stained gels. The amplification efficiencies were calculated using multiple dilutions of starting RNA, and reported results were

Supporting References

[1] Kemna EH, Tjalsma H, Podust VN, Swinkels DW. (2007) Mass spectrometry-based hepcidin measurements in serum and urine: analytical aspects and clinical implications. Clin Chem 53: 620-628.

[2] Zhou XY, Tomatsu S, Fleming RE, Parkkila S, Waheed A, Jiang J, et al. (1998) HFE gene knockout produces mouse model of hereditary hemochromatosis. Proc Natl Acad Sci USA 95: 2492-2497.

[3] Fleming RE, Ahmann JR, Migas MC, Waheed A, Koeffler HP, Kawabata H, et al. (2002) Targeted mutagenesis of the murine transferrin receptor-2 gene produces hemochromatosis. Proc Natl Acad Sci USA 99: 10653-10658.
